# Prevalence and correlates of postabortion long-acting reversible contraceptive (LARC) use among young women (24 and below) in Nepal: Strategy in the search for improvements

**DOI:** 10.1186/s12978-019-0708-7

**Published:** 2019-05-14

**Authors:** Suresh Mehata, Navaraj Bhattarai, Jamie Menzel, Mukta Shah, Pratik Khanal, Shadie Tofigh, Mukti Nath Khanal, Shibesh Chandra Regmi, Kathryn Andersen

**Affiliations:** 1 Ipas Nepal, Baluwatar, Do Cha Marg, Ward No.: 04, Kathmandu, 44600 Nepal; 2 Ipas, P.O. Box 9990, Chapel Hill, NC 27515 USA; 3 Ministry of Health, Ram Shah Path, Kathmandu, 44600 Nepal; 4 Institute of Medicine, Kathmandu, Nepal

**Keywords:** Long-acting reversible contraceptives, Induced abortion, Postabortion care, Health management information system, Nepal

## Abstract

**Introduction:**

Postabortion contraceptive use differs across countries, suggesting the need for country-level research to identify barriers and suggest appropriate interventions. This study aimed to identify the prevalence and correlates of postabortion long-acting reversible contraceptive (LARC) use among women aged 24 or younger in Nepal.

**Methods:**

This is a cohort study using Health Management Information System (HMIS) data where individual case records of women seeking induced abortion or postabortion care were documented using structured HMIS 3.7 records. Analysis was performed on the individual case records of 20,307 women 24 years or younger who received induced abortion or postabortion care services in the three-year period from July 2014 to June 2017 at 433 public and private health facilities.

**Findings:**

Overall, LARC uptake during the study period was 11% (IUD: 3% and implant: 8%). The odds of LARC acceptance was higher for young women (24 and below) who belonged to Brahmin/Chhetri (AOR = 1.23; 95% CI: 1.02–1.47) and Janajatis (AOR = 1.20; 95% CI: 1.01–1.43) as compared to Dalits; young women who had an induced abortion (AOR = 3.75; 95% CI: 1.75–8.06) compared with postabortion care; and those receiving service from public sector health facilities (AOR = 4.00; 95% CI: 2.06–7.75) compared with private sector health facilities.

**Conclusion:**

The findings from this study indicate the need to focus on barriers to acceptance of LARC among several groups of young women (24 and below) receiving abortion care in Nepal: Dalits, Madhesis and Muslims; nulliparous women; and those receiving services at private sector health facilities.

## Plain English summary

Long-acting reversible contraceptives (LARCs), offer the highest level of reversible protection against pregnancy and are safe, effective, and considered the most cost-effective methods. However, multiple barriers prevent their use in Nepal and result in unintended pregnancies.

This study utilizes data from HMIS 3.7 to assess the prevalence of LARC use and its association with age, education, number of living children, caste/ethnicity, gestational age, diagnosis, abortion method, facility type and sector, and postabortion contraceptive acceptance. Individual case records of women 24 years and younger receiving induced abortion or postabortion care were collected from 433 Ipas-supported health facilities between July 2014 and June 2017.

During the three-year period, 20,307 abortion services were provided to women aged 24 years and younger, 79% accepted a modern method of contraception. Most young women accepted short-acting contraceptive methods (68%) followed by LARC methods (11%) and permanent methods (0.4%).

The findings revealed that the use of LARC methods were higher among women who had had induced abortion and who had services from public sector health facilities. Also, young clients of the Brahmins/Chhetris and Janajatis ethnic groups were more likely to use LARCs as compared with the Dalits ethnic group.

In order to increase LARC use and reduce unintended pregnancy, the service should be available and accessible to young women at all facility levels with provision of quality counseling services. Additionally, focus should be given to young women who have had postabortion care, receive services from the private sector and those belonging to specific ethnic groups.

## Background

Postabortion contraceptive use is one of the essential elements of comprehensive abortion care to prevent future unwanted pregnancies and abortions [1]. Provision of abortion and postabortion contraception helps women end current and prevent future unintended pregnancies during the same visit. All women seeking abortion services should receive counseling on a range of contraceptive methods including short-acting, long-acting reversible and non-reversible methods to ensure women are offered methods that best meet their reproductive health needs. Long-acting reversible contraceptives (LARCs), such as intrauterine devices (IUDs) and sub-dermal implants, offer the highest level of reversible protection against pregnancy and for a longer time period [3 years to 12 years] [2, 3]. They are safe, effective, and considered the most cost-effective methods [3, 4].

The use of LARCs among women of reproductive age varies across the globe. In 2012, the percentage distribution of IUD users among women using modern contraceptive methods was 38% in high-income countries and 28% in developing countries [5]. At the country level, LARC use among postabortion clients is below 10% in Ghana [6], India [7], and Nepal [8, 9] and below 50% in Australia [10], New Zealand [11, 12], Sweden [13], and the USA [14]. Despite global evidence demonstrating the effectiveness of LARC methods, the low uptake among postabortion clients is a concern for policy makers and program managers. Evidence from different regions of the world suggest a number of inter-related factors such as demography, socioeconomics, awareness, provision of counseling services, health insurance, supplies, and health system capacity are responsible for low use of LARC methods [2, 10, 15–17]. Factors affecting LARC use differ between countries suggesting the need for country-level research to identify barriers to use [6–9].

Health services in Nepal are delivered at the primary, secondary and tertiary levels. The primary level consists of health posts, primary health care center (PHCCs) and outreach clinics. Secondary level services are delivered by district hospitals which also act as referral points for primary level facilities. Tertiary level services are provided by zonal, sub-regional, regional and central hospitals. Abortion services are provided at selected health facilities across all levels. In the districts where Ipas Nepal works, it supports comprehensive abortion care training for service providers and provides logistical and technical support to health facilities to provide high quality abortion and postabortion care.

In Nepal, legalization of abortion took place in September 2002 and safe abortion service provision started in March 2004 [18, 19]. In line with the National Safe Abortion Policy 2002, safe abortion services for abortion before 13 weeks gestation were expanded to all districts in Nepal through selected public and private health institutions [20]. By 2016, comprehensive abortion care (which includes induced abortion; treatment of incomplete, missed and unsafe abortion; compassionate counseling; contraceptive services; provision of sexual and reproductive health services or referrals to accessible facilities; and community-service provider partnerships) was integrated into existing service delivery in all district hospitals and in half of the PHCCs. Abortion at or after 13 weeks gestation was also available in 29 public central and zonal level hospitals and private hospitals [21]. In 2010, the Government of Nepal undertook the policy to provide at least five different modern contraceptive methods, including LARC methods, at the health post level. Despite this government strategy, the use of LARCs is relatively low as compared to other modern contraceptive methods. A recent population-based nationally representative survey reports that the modern contraceptive prevalence rate among married women of reproductive age is 43% and LARC methods account for only 5% [19].

Multiple barriers prevent the use of contraceptive methods in Nepal and result in unintended pregnancies. Socio-cultural factors, women’s empowerment, low community demand, and lack of familiarity with contraceptive methods are client-related barriers, whereas availability of services, supplies and human resource capacity and low quality of contraceptive counseling have been identified as key health system barriers to contraceptive use in Nepal [9, 22, 23]. Moreover, resource constraints, policy and legal restrictions, lack of professional encouragement and incentives to health workers, and inadequate focus on youth are policy-related barriers to postabortion contraceptive use [1, 3, 24–28]. In Nepal, among women who have given birth under the age of 25, 18% reported the pregnancy to be unintended [19]. A further analysis of the Nepal Demographic and Health Survey 2016 data revealed that among women who received abortion services, about one-fifth of them were youth aged 15 to 24 years. Additionally, among currently married women, the unmet need for contraception is high among youth (33%) when compared with the national average (24%) [19]. Thus, there is a need for clear and disaggregated data on youth’s postabortion contraceptive use and empirical evidence to explore the factors which hinder the use of postabortion contraception to prevent unintended pregnancies. In this context, this study aimed to identify the prevalence and correlates of postabortion LARC use among young women (24 and below) in Nepal and ways we might intervene or key populations on which to focus interventions.

### Program description

Ipas Nepal, a non-governmental organization, has been a key partner of the Government of Nepal in improving access to and quality of safe abortion services for over a decade. Through this partnership, provision of medical abortion at or before 9 weeks gestation has been supported in 46 out of Nepal’s 77 districts and 29 hospitals were supported to provide abortion at or after 13 weeks gestation. Ipas Nepal provides holistic technical and programmatic support in safe abortion programming. In coordination with the National Health Training Center, Ipas Nepal supported the training of healthcare providers and training of trainers for both medical abortion at or before 9 weeks and medical induction and dilatation and evacuation for abortion at or after 13 weeks gestation. Training content included information on comprehensive abortion care including youth and gender topics. Training sites and curricula were strengthened by ensuring evidence-based and up-to-date clinical information and culturally appropriate training materials, job aids and model equipment were available. In addition, a balanced counseling strategy – use of an algorithm to assess women’s needs and tailor counseling – was integrated into service provider trainings to improve postabortion contraceptive counseling [29]. After each training, need-based clinical support was provided to the service providers by clinical mentors. Likewise, Ipas Nepal supported the strengthening of health facilities to ensure they were equipped to provide high-quality abortion services. Additionally, the Government of Nepal has adopted this training approach for safe abortion services.

## Methods

### Study setting

In Nepal, there are 123 public hospitals (all levels), 200 PHCCs and 3808 health posts. Safe abortion services are provided by select health facilities at each level from health post to the central level. Altogether, there are 1326 listed facilities including public and private that provide safe abortion services in Nepal. Among these, Ipas Nepal provided technical and programmatic support to 433 of the 1326 listed facilities (33%) in abortion and postabortion contraceptive care between July 2014 and June 2017 (Table 1).

**Table 1:** Distribution of health facilities including the facilities providing safe abortion services

Facility Type	Total Health Facilities	Total Listed Health Facilities	Total Facilities Included in This Study	Included as % of All Listed
Public Hospitals	123	88	43	49%
Public Primary Health Care Centers	200	175	99	57%
Public Health Posts	3808	703	283	40%
Private Health Facilities	1715	360	8	2%
Total	5846	1326	433	33%

### Data collection methods

This is a cohort study using Health Management Information System (HMIS) data where individual case records of women 24 years and younger receiving induced abortion or postabortion care (PAC) in 433 Ipas-supported health facilities between July 2014 and June 2017 were documented using a structured HMIS 3.7 record. HMIS is a government owned and implemented information management system to collect, verify, process and analyze health service data. The following HMIS 3.7 variables were used in this analysis: age, education, number of living children, caste/ethnicity, gestational age, diagnosis (induced or PAC), abortion method (medical or surgical), facility type and sector, and postabortion contraceptive acceptance. Ipas Nepal staff collected data monthly and entered details into Ipas’s monitoring and evaluation database. Data were imported into Stata 14 for validation and analysis.

### Data management and analysis

Age was categorized into less than 20 years and 20 to 24 years. Education level included never attended school, primary, secondary and higher education. Caste/ethnicity included Dalits (the castes who were formerly considered “untouchable” according to the Hindu varna system), Janajatis (the indigenous groups), Madhesi (the Terai/Madhesi people are native inhabitants of the flat southern region of Nepal), Muslims, and Brahmin/Chhetri (the upper castes). Facility type included health posts and primary health care centers versus hospitals. Abortion method included medical (abortion by misoprostol and mifepristone) and surgical (abortion by manual vacuum aspiration or dilatation and evacuation). Gestational age was categorized as ≤12 weeks and ≥13 weeks. Diagnosis categories included induced (provider induced procedure) and PAC (care for an incomplete abortion). The primary outcome of interest was receipt of a LARC method.

Prevalence was determined if women accepted LARC immediately or within 14 days after an induced abortion or PAC, as recorded in the HMIS 3.7. For the purpose of this analysis, missing data for contraceptive acceptance (1.3%) were treated conservatively as not receiving a contraceptive method.

Descriptive statistics were reported using frequencies and percentages. To assess correlates of LARC use, we explored adjusted associations between LARC use and age, education, number of living children, caste/ethnicity, gestational age, diagnosis, abortion method, facility level and sector. All unadjusted and adjusted analyses were conducted using survey logistic regressions clustering on facility.

## Results

### Use of postabortion contraception

**Fig. 1:**
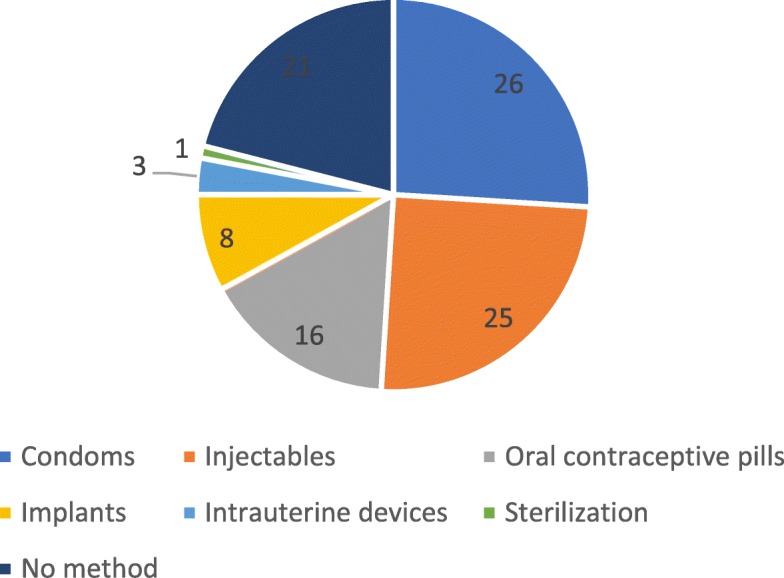
Contraceptive method mix among young women (24 and below) receiving abortion services in Ipas-supported facilities from July 2014 to June 2017 (*n* = 20,307)

**Fig. 2:**
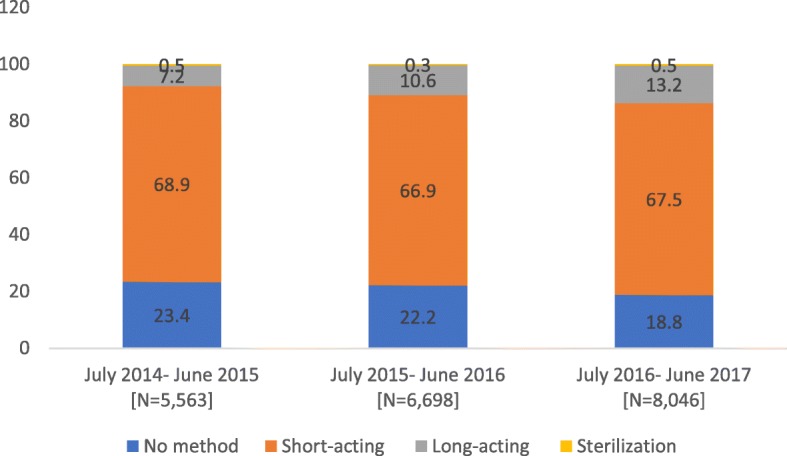
LARC use among young women (24 and below) increased yearly from July 2014 to June 2017 (*n* = 20,307)

During the three-year period, 20,307 abortion services were provided to women aged 24 years and younger, 79% accepted a modern method of contraception. Most young women (24 and below) accepted short-acting contraceptive methods (68%) followed by LARC methods (11%) and permanent methods (0.4%) (Fig. 1). The most preferred individual contraceptive method of choice was condoms (26%) followed by injectables (25%) and oral contraceptive pills (16%). For LARC methods, the use of implants (8%) was higher than the use of IUDs (3%). By fiscal year, LARC use almost doubled between July 2014 to June 2015 (7%) and July 2016 to June 2017 (13%) (Fig. 2).

### Prevalence of LARC use

The use of LARC methods was higher among women aged 20 to 24 years (12%) when compared with women below 20 years of age (7%). Use of LARCs did not vary much across education levels and ethnicity (Table 2). The greatest proportion of LARC use was among those who had primary education (12%), had three or more children (15%) and belonged to other ethnic groups (16%). The least use was among those with higher education (10%), had no children (3%) and belonged to the Muslim ethnic group (8%).

**Table 2:** Distribution of LARC use among young (24 and below) postabortion clients by socio-demographic characteristics and facility/service characteristics [*n* = 20,307]

	LARC Use		Total Clients
	N	%	
Client Age (years)			
< 20	251	6.5	3894
20–24	1930	11.8	16,413
Education level			
Never attended school	206	10.2	2014
Primary	284	12.4	2290
Secondary	987	10.7	9238
Higher education	476	9.8	4874
Missing	228	12.1	1891
Number of living children			
0	154	3.2	4789
1–2	1792	13.7	13,043
≥ 3	168	15.0	1123
Missing	67	5.0	1352
Caste/Ethnicity			
Dalit	279	9.9	2812
Janajati	832	10.6	7847
Madhesi	282	9.7	2903
Muslim	48	8.0	599
Brahmin/Chhetri	541	11.1	4871
Others^a^	107	15.6	686
Missing	92	15.6	589
Diagnosis			
Postabortion care	35	3.22	1088
Induced abortion	2146	11.2	19,219
Gestation			
≤ 12 weeks	2065	11.1	18,696
≥ 13 weeks	112	7.5	1487
Missing	4	3.2	124
Facility type			
HPs/PHCCs	1058	10.3	10,315
Hospitals	1123	11.2	9992
Abortion method			
Medical	1576	10.6	14,842
Surgical	605	11.1	5465
Facility sector			
Private	46	2.8	1633
Public	2135	11.4	18,674
Total	2181	10.7	20,307

^a^Others includes Thakuri and Sanyasi/Dashnami

Young women (24 and below) who had an induced abortion (11%) and those who had an abortion at or less than 12 weeks gestation (11%) were more likely to use LARCs than women who received PAC (3%) and had an abortion at or above 13 weeks (8%). Moreover, when analyzed across supply-side characteristics such as health facility sector and type, the use of LARCs was higher among women who visited public sector health facilities (11%) than those who visited private sector health facilities (3%) (Table 2).

### Correlates of LARC use

In the adjusted model, the odds of LARC acceptance was higher among clients who belonged to Brahmin/Chhetri (OR = 1.23; 95% CI: 1.02–1.47) and Janajatis (OR = 1.20; 95% CI: 1.01–1.43) caste as compared to Dalits, among those who sought induced abortion (OR = 3.75; 95% CI: 1.75–8.06) compared with postabortion care, and among those who received service from public sector health facilities (OR = 4.00; 95% CI: 2.06–7.75) compared with private sector health facilities (Table 3). Also, compared with young women (24 and below) who had no living children, a higher odds of LARC acceptance was observed among those who have one or two (OR = 4.59; 95% CI: 3.77–5.57) and three or more living children (OR = 5.52; 95% CI: 4.24–7.17).

**Table 3:** Correlates of LARC use among young women (24 and below) in Nepal [*n* = 20,307]

	Bivariate			Multivariate		
	OR	95% CI of OR	P	OR^a^	95% CI of OR	P
Client Age (years)						
< 20	Reference			Reference		
20–24	1.93	1.65–2.26	< 0.001	1.02	0.87–1.19	0.817
Education level						
Never attended school	Reference			Reference		
Primary	1.24	0.99–1.55	0.057	1.18	0.95–1.48	0.133
Secondary	1.05	0.85–1.29	0.645	1.08	0.90–1.30	0.412
Higher education	0.95	0.75–1.21	0.672	1.10	0.88–1.37	0.390
Number of living children						
0	Reference			Reference		
1–2	4.79	3.95–5.81	< 0.001	4.59	3.77–5.57	< 0.001
> 3	5.29	3.74–7.49	< 0.001	5.52	4.24–7.17	< 0.001
Caste/Ethnicity						
Dalit	Reference			Reference		
Janajati	1.08	0.87–1.33	0.491	1.20	1.01–1.43	0.034
Madhesi	0.98	0.74–1.28	0.865	0.98	0.75–1.29	0.907
Muslim	0.79	0.53–1.18	0.247	0.82	0.55–1.21	0.314
Brahmin/Chhetri	1.13	0.95–1.36	0.165	1.23	1.02–1.47	0.031
Others	1.68	1.14–2.47	0.009	1.68	1.45–2.35	< 0.001
Diagnosis						
Postabortion care	Reference			Reference		
Induced abortion	3.78	2.02–7.07	< 0.001	3.75	1.75–8.06	0.001
Gestation						
≤ 12 weeks	Reference			Reference		
≥ 13 weeks	0.66	0.45–0.96	0.028	0.84	0.67–1.05	0.134
Facility type						
HPs/PHCCs	Reference			Reference		
Hospitals	1.11	0.64–1.92	0.530	1.47	0.89–2.45	0.136
Abortion method						
Medical	Reference			Reference		
Surgical	1.05	0.65–1.68	0.845	1.15	0.90–1.49	0.267
Facility sector						
Private	Reference			Reference		
Public	4.45	2.68–7.41	< 0.001	4.00	2.06–7.75	< 0.001

^a^Adjusted odds ratios have been adjusted for all variables listed and clustering on facility

After adjusting, no significant association was observed with client age, education, gestational age, facility type and abortion method.

## Discussion

Overall, 79% of young women (24 and below) who received safe abortion services accepted a modern method of contraception. Short-acting contraceptive method use was 68% followed by LARC methods (11%) and permanent methods (0.4%). There was a higher proportion of the postabortion LARC users (16%) in a study conducted at the central level maternity hospital of Nepal [22], while LARC use was as low as 1.4% among married women of reproductive age (15–49 years) in one study in rural Nepal [30].

Evidence suggests that high-quality and client-centered counseling on contraception with a focus on modern contraceptives, including LARC, pre-procedure and during the postabortion period is effective to increase LARC use [1, 18, 25, 31]. However, the Nepal Demographic Health Survey 2016, revealed that only about half of the abortions (51%) nationally were conducted in authorized facilities. Likewise, about half of women aged 15–49 years (53%) received information on contraception during the postabortion period and only a quarter used a contraceptive method within two weeks of abortion services [19]. The health system in Nepal is further challenged with poor readiness to provide abortion and contraception services as evidenced by the findings of Nepal Health Facility Survey 2015; fewer than half of the health facilities provided LARC and only one out of four health workers were trained in postabortion care at any time [32]. The same survey showed that the quality of contraceptive counseling was poor with only 6% of health facilities maintaining privacy/confidentiality. Studies have shown that increasing awareness among clients about LARC and removing financial barriers increases LARC use [17]. Provision of high-quality postabortion contraceptive counseling services that focuses on increased knowledge along with availability of LARC at health facilities is thus necessary to increase the uptake of LARC.

Our findings did not suggest an association of postabortion LARC use with age among young women. This finding is not consistent with studies done in Australia [10] and in ten countries of Asia and Sub-Saharan Africa [18]. This might be due to the disinclination of providers to promote LARC use among all young clients (24 and below) coupled with low demand by young clients because of the fear of infertility [6, 33]. Young clients (24 and below) may also need special focus as they might have issues with access and cost [34]. Additionally, they might be less knowledgeable about LARC methods in general [35]. Studies have shown that poor women have low LARC uptake although the mechanisms of this inequity are not clearly established [10, 36, 37].

Interestingly, our study revealed that young women (24 and below) with higher education were significantly less likely to use a LARC method as compared with young women (24 and below) who never attended school. This might be because women with higher education are more likely to have better economic opportunities and opt for private facilities when seeking abortion services. In our study, young clients (24 and below) of the Brahmins/Chhetris ethnic groups (the dominant ethnic groups in Nepal) were significantly more likely to use LARCs as compared with the Dalits ethnic group. Similar studies from the United States [38] and New Zealand [12] also demonstrated ethnicity as a predictor of LARC use, although it was not the same in a study from India [39].

Our study shows young clients (24 and below) who received an induced abortion were nearly four times more likely to use a LARC method as compared with clients receiving PAC. While our findings may be affected by the low number of women receiving PAC, it is also natural that women who seek postabortion care after having a spontaneous abortion are likely to want to get pregnant again soon and may not demand LARCs. Another possible reason providers are reluctant to offer LARC methods is due to fear of infections [40] and prioritizing immediate medical needs rather than postabortion contraceptive counseling [18].

Our findings reveal that clients visiting public sector facilities as compared with private sector facilities are more likely to accept a LARC method. One reason behind this result may be due to better availability of contraceptive supplies and trained providers in public facilities and relatively poor linkages between abortion and contraceptive services in private facilities [18]. The Government of Nepal and all stakeholders should make efforts to ensure that there is availability and provision of LARC at all safe abortion sites. Studies have shown that expanding contraceptive method choice and availability of services for immediate LARC insertion following abortion increased LARC use [26, 41–43]. In our study, method of abortion was not associated with LARC use. This finding was inconsistent with a study done in Australia [10] where after surgical abortion women had a significantly higher LARC preference as compared with medical abortion.

The findings from this study imply that a greater focus needs to be placed on women receiving postabortion care; those who belong to Madhesi, Muslim and Dalit caste groups; nulliparous women and addressing issues with low use in private sector and primary health facilities. Taking into account the evidence from other countries, improving access, focusing on quality of care, integration of reproductive health services, timely LARC provision, improving referral systems and follow-up mechanisms of clients, improving community-service provider linkages, use of information systems in decision making, broader method mix and arrangements for youth and culture friendly services and partner communication might improve LARC use in Nepal [1, 7, 10, 14, 24, 26, 37, 43].

Our study has some limitations as well. We could not assess other factors such as access to and cost of LARC, continuation of LARC methods, method availability at health facilities and trained providers, quality of contraceptive counseling services, place of residence, client familiarity with LARCs, involvement of partners in contraceptive decision making, and the socio-economic status of women which could have affected LARC use. Assessing the role of such factors with LARC use could be further areas of research. Despite its limitations, this study helps to characterize postabortion contraceptive use with a focus on LARC among young women seeking abortion care at Ipas-supported facilities in Nepal.

## Conclusion

Among young women (24 and below) receiving abortion services, the proportion accepting a postabortion LARC method was 11%. Predictors of LARC uptake were ethnicity, abortion diagnosis, and health facility sector. With global evidence suggesting the effectiveness of LARCs in reducing unintended pregnancies, family planning strategies should promote modern contraceptives including LARC; ensuring that these services are available and accessible to young women (24 and below) at all facility levels with provision of high-quality counseling services. Health workers should be equipped with skills to provide quality contraceptive counseling and services that account for specific needs of young women and consider the diversity among youth and strengthen linkages between contraceptive and abortion care at public and private facilities.

## References

[1] Corbett MR, Turner KL (2003). Essential elements of postabortion care: origins, evolution and future directions. Int Fam Plan Perspect.

[2] Stanek AM, Bednarek PH, Nichols MD, Jensen JT, Edelman AB (2009). Barriers associated with the failure to return for intrauterine device insertion following first-trimester abortion. Contraception.

[3] Thompson KM, Speidel JJ, Saporta V, Waxman NJ, Harper CC (2011). Contraceptive policies affect post-abortion provision of long-acting reversible contraception. Contraception.

[4] Winner B, Peipert JF, Zhao Q, Buckel C, Madden T, Allsworth JE, Secura GM (2012). Effectiveness of long-acting reversible contraception. N Engl J Med.

[5] Darroch JE, Singh S (2013). Trends in contraceptive need and use in developing countries in 2003, 2008, and 2012: an analysis of national surveys. Lancet.

[6] Rominski SD, Morhe ES, Lori J (2015). Post-abortion contraception choices of women in Ghana: a one-year review. Global Public Health.

[7] Zavier A, Padmadas SS (2012). Postabortion contraceptive use and method continuation in India. Int J Gynecol Obstet.

[8] Puri M, Henderson JT, Harper CC, Blum M, Joshi D, Rocca CH (2015). Contraceptive discontinuation and pregnancy postabortion in Nepal: a longitudinal cohort study. Contraception.

[9] Rocca CH, Puri M, Harper CC, Blum M, Dulal B, Henderson JT (2014). Postabortion contraception a decade after legalization of abortion in Nepal. Int J Gynecol Obstet.

[10] Goldstone P (2014). Factors predicting uptake of long-acting reversible methods of contraception among women presenting for abortion. America (North and South).

[11] Rose SB, Garrett SM (2015). Post-abortion initiation of long-acting reversible contraception in New Zealand. J Fam Plann Reprod Health Care.

[12] Rose SB, Garrett SM, Stanley J (2015). Immediate postabortion initiation of levonorgestrel implants reduces the incidence of births and abortions at 2 years and beyond. Contraception.

[13] Kilander H, Alehagen S, Svedlund L, Westlund K, Thor J, Brynhildsen J (2016). Likelihood of repeat abortion in a Swedish cohort according to the choice of post-abortion contraception: a longitudinal study. Acta Obstet Gynecol Scand.

[14] Stacey RE, Dempsey AR (2014). The influence of trust in health care systems on postabortion contraceptive choice. Obstet Gynecol.

[15] Gudaynhe SW, Zegeye DT, Asmamaw T, Kibret GD. Factors Affecting the use of Long-Acting Reversible Contraceptive Methods among Married Women in Debre Markos Town, Northwest Ethiopia 2013. Glob J Med Res. 2014;14(5):Version 1.0.

[16] Kabalo MY (2016). Utilization of reversible long acting family planning methods among married 15-49 years women in Areka town, southern Ethiopia. Int J Sci Rep.

[17] Secura GM, Allsworth JE, Madden T, Mullersman JL, Peipert JF (2010). The Contraceptive CHOICE Project: reducing barriers to long-acting reversible contraception. Am J Obstetr Gynecol.

[18] Benson J, Andersen K, Healy J, Brahmi D (2017). What factors contribute to Postabortion contraceptive uptake by young women? A program evaluation in 10 countries in Asia and sub-Saharan Africa. Global Health Sci Pract.

[19] Ministry of Health NNEaI: Nepal Demographic and Health Survey 2016. In. Kathmandu, Nepal; 2017.

[20] MoH: National Safe Abortion Policy 2002. In. Edited by FHD D. Kathmandu, Nepal: FHD, DoHS; 2002.

[21] MoH: Annual Report 2015/16. In. Kathmandu, Nepal: Department of Health Services; 2016.

[22] Paudel P, Paudel L, Bhochhibhoya M, Vaidhya SA, Shah N, Khatiwada D. Pattern of abortion care in a tertiary level maternity hospital in Nepal. J Nepal Med Assoc. 2013:52(191). 24907945

[23] Wang L-F, Puri M, Rocca CH, Blum M, Henderson JT (2016). Service provider perspectives on post-abortion contraception in Nepal. Cult Health Sex.

[24] Mazza D, Bateson D, Frearson M, Goldstone P, Kovacs G, Baber R (2017). Current barriers and potential strategies to increase the use of long-acting reversible contraception (LARC) to reduce the rate of unintended pregnancies in Australia: an expert roundtable discussion. Aust N Z J Obstet Gynaecol.

[25] Borges ALV, OlaOlorun F, Fujimori E, Hoga LAK, Tsui AO (2015). Contraceptive use following spontaneous and induced abortion and its association with family planning services in primary health care: results from a Brazilian longitudinal study. Reprod Health.

[26] Samuel M, Fetters T, Desta D (2016). Strengthening postabortion family planning services in Ethiopia: expanding contraceptive choice and improving access to long-acting reversible contraception. Glob Health Sci Pract.

[27] Mugore S, Kassouta NTK, Sebikali B, Lundstrom L, Saad A (2016). Improving the quality of Postabortion Care Services in Togo Increased Uptake of contraception. Glob Health Sci Pract.

[28] Morse J, Freedman L, Speidel JJ, Thompson KM, Stratton L, Harper CC (2012). Postabortion contraception: qualitative interviews on counseling and provision of long-acting reversible contraceptive methods. Perspect Sex Reprod Health.

[29] Farrokh-Eslamlou H, Aghlmand S, Khorasani-Zavareh D, Mohammad Alizadeh Charandabi S, Moghaddam Tabrizi F, Jahanfar S. Structured versus routine family planning counselling for contraception. Cochrane Database Syst Rev. 2014;7.

[30] Mehata Suresh, Paudel Yuba Raj, Dotel Bhogendra Raj, Singh Dipendra Raman, Poudel Pradeep, Barnett Sarah (2014). Inequalities in the Use of Family Planning in Rural Nepal. BioMed Research International.

[31] Whitaker AK, Quinn MT, Munroe E, Martins SL, Mistretta SQ, Gilliam ML (2016). A motivational interviewing-based counseling intervention to increase postabortion uptake of contraception: a pilot randomized controlled trial. Patient Educ Couns.

[32] Nepal NE: Nepal: health facility survey 2015. Nepal: health facility survey 2015 2017.

[33] Rominski SD, Lori JR (2014). Abortion care in Ghana: a critical review of the literature. Afr J Reprod Health.

[34] Nalwadda G, Mirembe F, Tumwesigye NM, Byamugisha J, Faxelid E (2011). Constraints and prospects for contraceptive service provision to young people in Uganda: providers' perspectives. BMC Health Serv Res.

[35] Hoopes A, Ahrens K, Gilmore K, Cady J, Haaland W, Amies-Oelschlager AM, Prager S (2015). Knowledge and attitudes about long-acting reversible contraception among female school-based health center patients: a pilot survey. Contraception.

[36] Keene M, Roston A, Keith L, Patel A (2015). Effect of previous induced abortions on postabortion contraception selection. Contraception.

[37] Rose SB, Cooper AJ, Baker NK, Lawton B (2011). Attitudes toward long-acting reversible contraception among young women seeking abortion. J Women's Health.

[38] Kavanaugh ML, Carlin EE, Jones RK (2011). Patients' attitudes and experiences related to receiving contraception during abortion care. Contraception.

[39] Paul M, Iyengar SD, Essén B, Gemzell-Danielsson K, Iyengar K, Bring J, Klingberg-Allvin M (2016). Does mode of follow-up influence contraceptive use after medical abortion in a low-resource setting? Secondary outcome analysis of a non-inferiority randomized controlled trial. BMC Public Health.

[40] Banerjee S, Gulati S, Andersen K, Acre V, Warvadekar J, Navin D (2015). Associations between abortion services and acceptance of Postabortion contraception in six Indian states. Stud Fam Plan.

[41] Madden T, Secura GM, Allsworth JE, Peipert JF (2011). Comparison of contraceptive method chosen by women with and without a recent history of induced abortion. Contraception.

[42] Madden T, Eisenberg DL, Zhao Q, Buckel C, Secura GM, Peipert JF (2012). Continuation of the etonogestrel implant in women undergoing immediate postabortion placement. Obstet Gynecol.

[43] Sääv I, Stephansson O, Gemzell-Danielsson K (2012). Early versus delayed insertion of intrauterine contraception after medical abortion—a randomized controlled trial. PLoS One.

